# An evaluation of DeepSeek and healthcare professionals’ Q&A capabilities in improving patient-family satisfaction in the ICU

**DOI:** 10.3389/fmed.2026.1859158

**Published:** 2026-06-08

**Authors:** Min Li, Ruo-Yu Li, Wei-Jie Lai, Feng-Ling Yang, Cui-Bing Liu

**Affiliations:** 1 Department of Critical Care Medicine, Dongguan Hospital of Guangzhou University of Chinese Medicine (Dongguan Hospital of Traditional Chinese Medicine), Dongguan, Guangdong, China; 2 Department of Critical Care Medicine, The Sixth Affiliated Hospital of Guangdong Pharmaceutical University, Dongguan, Guangdong, China

**Keywords:** artificial intelligence, intensive care unit, large language models, questions and answers, short assessment of patient satisfaction

## Abstract

**Background:**

Families of intensive care unit (ICU) patients often require timely medical updates, creating additional communication demands for clinicians under heavy workloads. Large language models (LLMs), such as DeepSeek, may help support clinician–family communication, but their quality and reliability require empirical evaluation.

**Methods:**

Two ICU clinicians with more than 10 years of experience developed 30 representative questions reflecting common consultation scenarios encountered by family members of ICU patients. Responses were independently generated by clinician and DeepSeek-V3.1. Medical accuracy, information completeness, clarity and empathy, and safety assessment were evaluated by recruited simulated patients and ICU physicians, while satisfaction was evaluated by 600 family members of ICU patients.

**Results:**

DeepSeek showed significantly better performance than clinicians in information completeness, clarity and empathy, and patient-family satisfaction (*p* < 0.05). In the DeepSeek group, 66.7% of family members reported being satisfied with the responses. Mixed-effects ordinal logistic regression further confirmed that DeepSeek had higher odds of receiving better ratings in information completeness, clarity and empathy, and patient-family satisfaction, while no significant differences were observed in medical accuracy or safety assessment. These findings indicate that DeepSeek performed better mainly in communication-related domains while maintaining comparable accuracy and safety under standardized written evaluation conditions.

**Conclusion:**

DeepSeek provided more complete, clear, and empathetic written responses to standardized ICU family consultation questions, with higher perceived family satisfaction than clinician responses. These findings suggest that LLMs may support ICU communication, but clinician oversight remains essential for individualized, prognostic, or safety-sensitive discussions. Real-world studies are needed to confirm their safety, reliability, and clinical applicability.

## 1 Introduction

The Intensive Care Unit (ICU) is a highly specialized setting designed to provide advanced care for critically ill patients. It features state-of-the-art monitoring systems and a multidisciplinary team that delivers continuous, individualized treatment (1). Nevertheless, the complexity of the ICU environment and the severity of patients’ conditions often contribute to substantial psychological and emotional stress (2, 3). Thus, beyond medical interventions, healthcare providers must also address patients’ emotional and social needs.

In recent years, patient satisfaction has emerged as a key indicator of healthcare quality (4–6). As a direct reflection of patients’ perceptions, satisfaction affects important outcomes, including healthcare utilization, adherence to treatment, and the stability of the clinician-patient relationship (7, 8). Effective communication is central to achieving higher satisfaction, as transparent explanations and clear discussions of medical conditions can alleviate anxiety, prevent misunderstandings, and improve compliance and overall satisfaction (9–11).

ICU patients frequently encounter rapidly evolving and complex clinical scenarios, prompting repeated inquiries from patient-family members (12). This imposes substantial communication demands on clinicians, adding to their already heavy workload (13). Moreover, with the rise of digital health technologies and broader access to medical information, patient-family members increasingly expect more detailed, personalized explanations from healthcare providers (14).

Amid these challenges, recent advances in artificial intelligence (AI), particularly large language models (LLMs), may help improve healthcare communication. LLMs have demonstrated notable capabilities in natural language understanding and generation (15), with applications including postoperative rehabilitation guidance, health consultations, and patient management (16, 17). Evidence suggests that AI-driven systems can alleviate clinicians’ communication burden while providing timely, accurate, and comprehensible information, thereby improving patient satisfaction (18). Beyond these applications, LLMs are increasingly being explored in patient education and clinician-patient communication. Their ability to explain complex medical information in plain language may help patients and families better understand disease conditions, treatment plans, and care-related decisions (19, 20). Recent studies have reported that AI-generated responses to patient questions were rated favorably in terms of quality and empathy compared with physician responses (21). AI-generated draft replies to patient portal messages have also been introduced in clinical settings to help clinicians manage growing communication demands. However, issues related to accuracy, safety, accountability, and workflow integration remain important concerns (22, 23).

Accordingly, this study aims to compare the performance of the DeepSeek model with that of experienced ICU clinicians in responding to inquiries from patient-family members. It also evaluates the impact of AI-assisted communication on patient-family satisfaction, providing empirical support for the safe and effective integration of AI into critical care practice.

## 2 Methods

This study was approved by the Medical Ethics Committee of Dongguan Hospital of Traditional Chinese Medicine (Approval No. PJ-2025-131). Written informed consent was obtained from all participants prior to enrollment. All research procedures were conducted in strict accordance with applicable ethical guidelines to ensure the confidentiality and privacy of participant information. The CHART checklist is provided in Supplementary Table 1.

### 2.1 Study design

**Figure 1:**
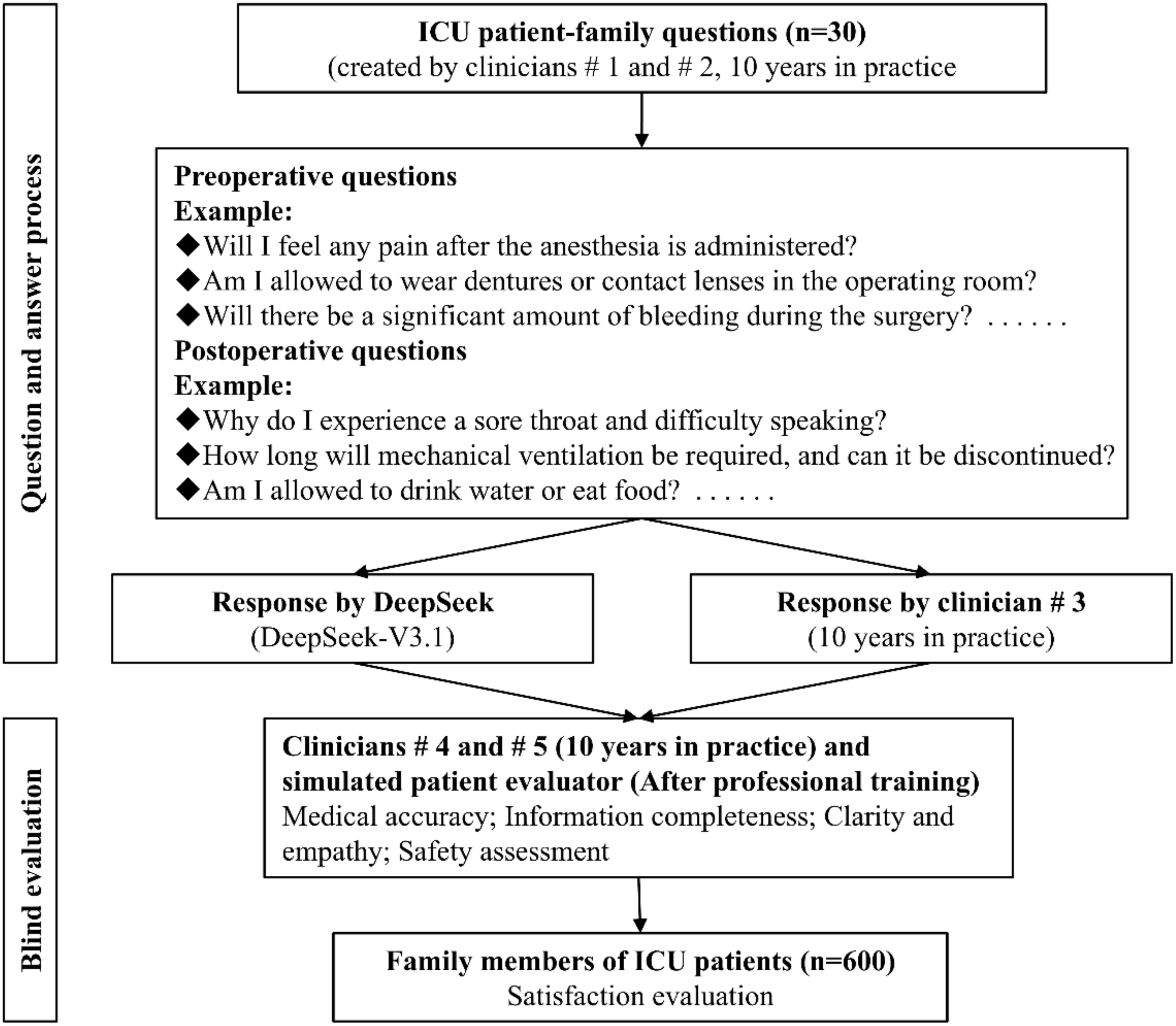
Overview of the study design.

This study was designed as a standardized vignette-based evaluation rather than a real-world clinical communication intervention. Figure 1 illustrates the overall study design. Specifically, 30 predefined ICU-related patient-family questions were developed in advance and used as standardized prompts to compare responses generated by DeepSeek and clinicians under the same conditions. Thus, the study assessed the quality of written responses to a fixed question bank rather than patient-specific, real-time communication in actual clinical care.

The 30 standardized questions were developed in Chinese by two ICU clinicians, each with more than 10 years of experience, to reflect common ICU family consultation scenarios. To capture real-world communication needs, the clinicians reviewed topics frequently encountered during ICU family meetings, bedside updates, and telephone inquiries. These topics covered disease severity and prognosis, treatment plans, life-support measures, clinical changes, medications and procedures, nutrition and rehabilitation, infection control, financial or care-related concerns, and emotional support. Candidate questions were selected if they reflected common family concerns, applied to a broad range of critically ill patients, required clear medical explanation, and supported family understanding, decision-making, or reassurance. The question set also included perioperative and anesthesia-related critical care questions but was not intended to cover complex discussions such as end-of-life care, goals of care, or long-term outcomes.

After the initial list was generated, the two clinicians refined the wording of each question to improve clarity, avoid duplication, and ensure that the questions were understandable from the perspective of non-medical family members. Questions that were too disease-specific, overly technical, or dependent on individual patient data were excluded, because the purpose was to evaluate responses to standardized and broadly representative ICU communication scenarios. The final 30 questions were reviewed by another ICU clinician with 10 years of experience to confirm their clinical relevance, representativeness, and suitability for standardized evaluation. The complete set of questions is provided in Supplementary Table 2.

Each question was submitted in the same written format to both the freely accessible DeepSeek-V3.1 model (September 22, 2025) and a single clinician for independent response generation (24). All response-generation sessions were conducted through the DeepSeek web-based platform in separate chat sessions at the Clinical Research Center of Dongguan Traditional Chinese Medicine Hospital on October 16, 2025. To ensure consistency, all prompts were prepared with reference to authoritative medical knowledge bases and current evidence and were reviewed by an ICU clinician with 10 years of experience before use (25, 26).

The generated responses were anonymized and evaluated using predefined scoring criteria. Before evaluation, all responses were stripped of source-identifying information, assigned anonymous codes, and randomly ordered. Evaluators were not informed whether each response was generated by DeepSeek or by clinicians. Medical accuracy, information completeness, clarity and empathy, and safety assessment were evaluated by recruited simulated patients and physicians according to predefined scoring criteria. Satisfaction was evaluated by 600 family members of ICU patients, all of whom were blinded to the response source. These participants served as evaluators of standardized responses rather than recipients of communication regarding their own relatives.

A total of 600 family members of ICU patients were recruited for the satisfaction assessment. The inclusion criteria were as follows: (1) adult patients over 18 years of age; (2) being a first-degree relative or primary caregiver of the patient; and (3) ability to independently read, understand, and complete the study assessments. The exclusion criteria were as follows: (1) cognitive impairment or other conditions preventing independent completion of the assessments; (2) refusal to participate or withdrawal during the study; and (3) enrollment of another family member of the same patient. Baseline characteristics of patients and their family members are summarized in Supplementary Table 3.

### 2.2 Outcome assessment

The primary outcome was patient-family satisfaction with responses generated by clinicians and DeepSeek, assessed using the Short Assessment of Patient Satisfaction (SAPS) scale (27). SAPS scores ranged from 1 (very dissatisfied) to 5 (very satisfied), with higher scores indicating greater satisfaction. Satisfaction was evaluated independently by participating family members of ICU patients, who were blinded to the source of the responses.

Secondary outcomes included four domains related to response quality and safety: medical accuracy, information completeness, clarity and empathy, and safety. Each domain was assessed using a 5-point Likert scale, with higher scores indicating better performance. Medical accuracy was rated from 1 = serious errors to 5 = fully accurate; information completeness from 1 = severely deficient to 5 = very comprehensive; clarity and empathy from 1 = unclear and impersonal to 5 = highly clear and empathetic; and safety from 1 = highly dangerous to 5 = very safe. Before formal evaluation, all assessors received standardized instructions regarding the study purpose, definitions of each scoring domain, and the application of the scoring criteria. Representative examples were used to promote a consistent interpretation of the rating scale.

Medical accuracy, information completeness, clarity and empathy and safety were assessed jointly by simulated family-member actors and two independent ICU physicians with more than 10 years of clinical experience who were not involved in question development or response generation. The physicians focused on the correctness of medical information, the adequacy of key ICU-related explanations, and the presence of potentially misleading or unsafe recommendations. Professionally trained simulated family-member actors evaluated clarity and empathy from the perspective of non-medical family members. They were trained to assess whether the responses were understandable, respectful, supportive, and responsive to the emotional concerns of families of critically ill patients. To reduce assessment bias, all responses were anonymized, randomly ordered, and presented without identifying whether they were generated by clinicians or DeepSeek. All assessors, including ICU physicians, simulated family-member actors, and family-member evaluators, were blinded to the response source throughout the evaluation process. The same scoring framework was applied to both groups to ensure comparability.

### 2.3 Statistical analyses

Continuous variables are presented as mean ± standard deviation for normally distributed data and as median (interquartile range) for non-normally distributed data. Categorical variables are presented as frequencies and percentages. Because responses from DeepSeek and clinicians were generated for the same set of standardized questions, paired between-group comparisons at the question level were performed using the Wilcoxon signed-rank test. Proportions were compared using z-tests, and 95% confidence intervals were reported where appropriate. To further account for the non-independence of ratings arising from repeated evaluations of the same standardized questions, a mixed-effects ordinal logistic regression analysis was performed for each evaluation domain. In this model, response source (DeepSeek versus clinician) was included as the fixed effect, and question item was included as a random effect. All statistical tests were two-sided, and *p* values < 0.05 were considered statistically significant. Statistical analyses were performed using IBM SPSS Statistics version 27 (IBM Corp., Armonk, NY, USA).

## 3 Results

### 3.1 DeepSeek

**Figure 2:**
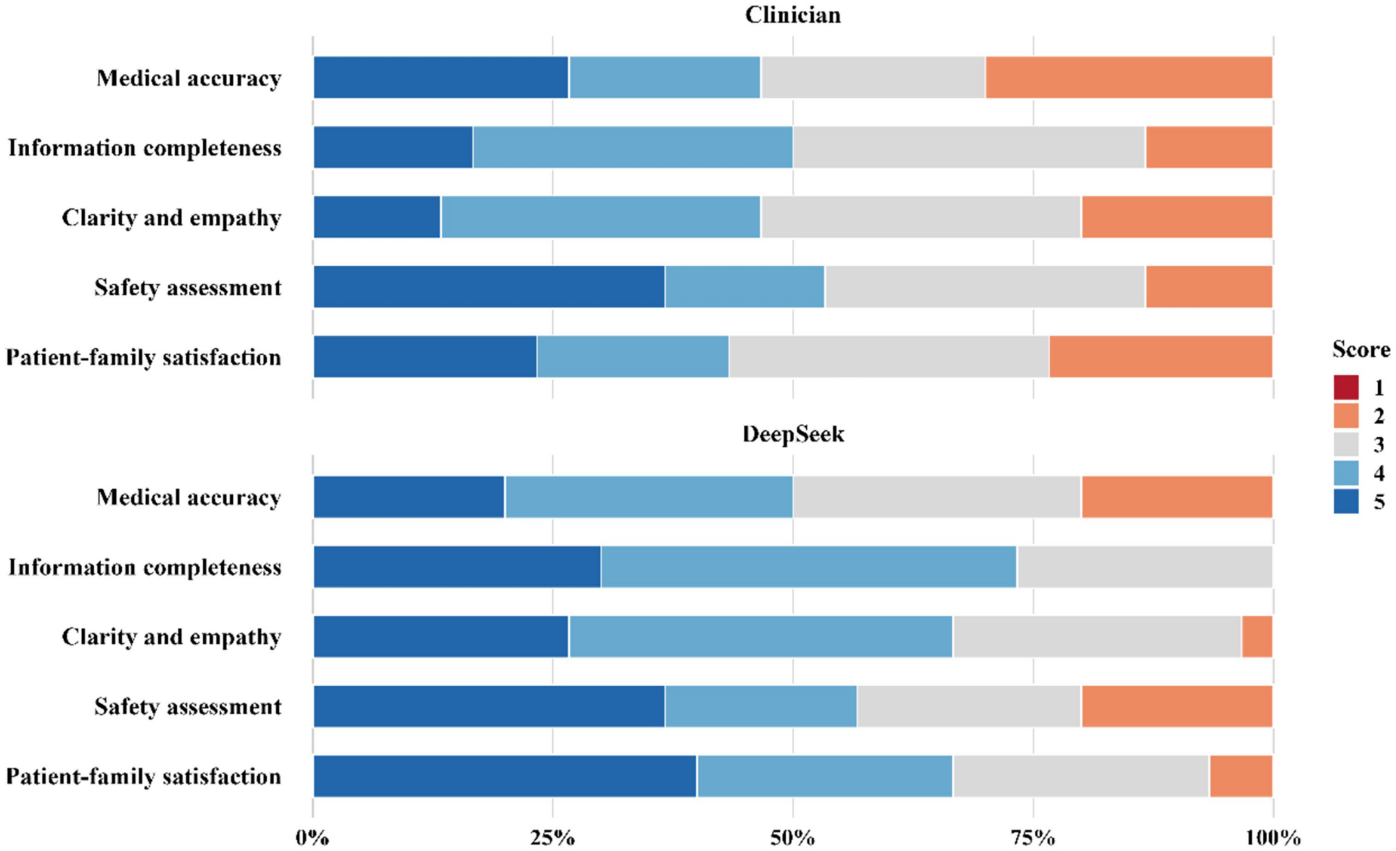
Likert scale stacked chart.

DeepSeek responses were evaluated across five domains: medical accuracy, information completeness, clarity and empathy, safety, and patient-family satisfaction. As shown in Figure 2 and Tables 1, 2, DeepSeek generally demonstrated a favorable distribution of ratings across all domains, with a clear tendency toward higher-category ratings in information completeness, clarity and empathy, and patient-family satisfaction. Ratings for medical accuracy and safety were more moderate, although the overall distribution remained acceptable.

**Table 1:** DeepSeek vs. clinician score summary.

Evaluation project	DeepSeek	Clinician	*P*-value
Medical accuracy	3.5 (3.0–4.0)	3.0 (2.0–4.8)	0.801
Information completeness	4.0 (3.3–5.0)	3.5 (3.0–4.0)	0.037
Clarity and empathy	4.0 (3.0–4.8)	3.0 (3.0–4.0)	0.047
Safety assessment	4.0 (3.0–5.0)	4.0 (3.0–5.0)	0.920
Patient-family satisfaction	4.0 (3.0–5.0)	3.0 (3.0–4.0)	0.044

Continuous non-normal data are presented in median (interquartile ranges).

**Table 2:** Scoring overview.

Evaluation project	DeepSeek		Clinician	
	Positive attitude	Highest proportion	Positive attitude	Highest proportion
Medical accuracy	15 (50.0%)	4 (30.0%)	14 (46.7%)	2 (30.0%)
Information completeness	22 (73.3%)	4 (43.3%)	15 (50.0%)	3 (36.7%)
Clarity and empathy	20 (66.7%)	4 (40.0%)	14 (46.7%)	3 (33.3%)
Safety assessment	17 (56.7%)	5 (36.7%)	16 (53.3%)	5 (36.7%)
Patient-family satisfaction	400 (66.7%)	5 (40.0%)	260 (43.3%)	3 (33.3%)

Categorical variables are presented in frequency (%); A positive attitude is defined as a score of 4 or higher.

### 3.2 Clinician

Clinician responses were also assessed across the same five domains. Compared with DeepSeek, clinician responses showed a greater concentration in the mid-range rating categories, particularly for information completeness, clarity and empathy, and patient-family satisfaction. In contrast, the distribution patterns for medical accuracy and safety were broadly similar to those observed for DeepSeek.

### 3.3 DeepSeek vs. clinician

**Figure 3:**
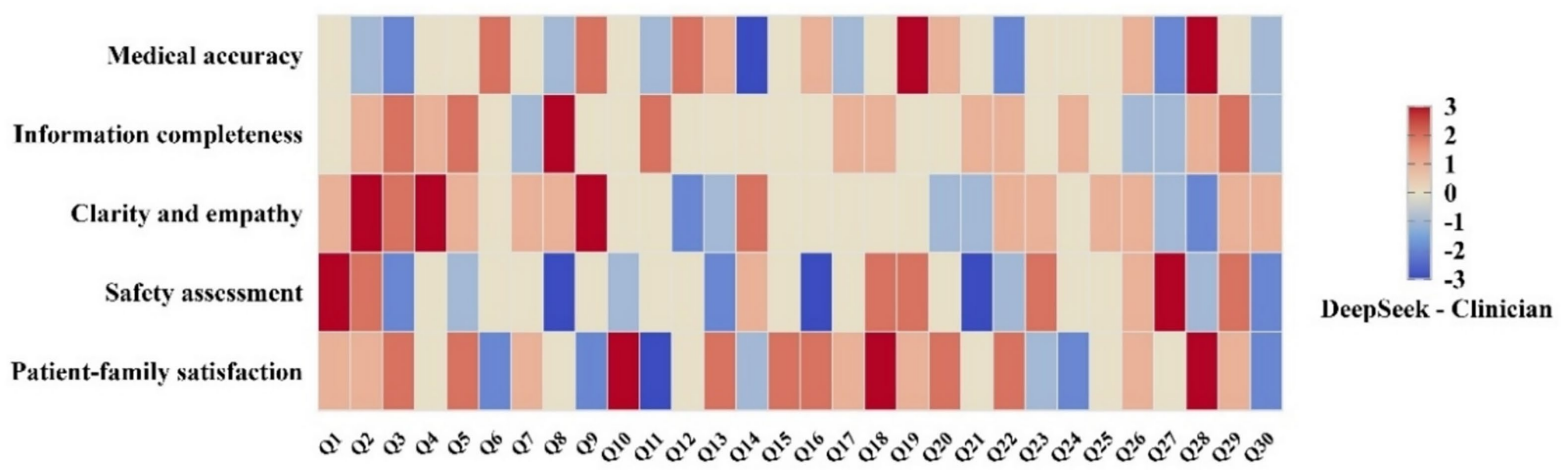
Heatmap of score differences between DeepSeek and clinician responses across 30 standardized ICU family questions.

As summarized in Table 1, DeepSeek achieved significantly higher median scores than clinicians in information completeness (*p* = 0.037), clarity and empathy (*p* = 0.047), and patient-family satisfaction (*p* = 0.044). No significant differences were found in medical accuracy (*p* = 0.801) or safety assessment (*p* = 0.920). Consistent with these findings, the stacked Likert bar charts showed that DeepSeek responses were more frequently rated in the higher score categories (4–5), whereas clinician responses were more often concentrated in intermediate categories. Overall, these results suggest that DeepSeek provided more complete, clearer, and more empathetic responses and achieved higher satisfaction ratings, while maintaining comparable accuracy and safety. To further examine item-level differences, a heatmap was used to compare score differences between DeepSeek and clinician responses across the 30 standardized ICU family questions and five evaluation domains. DeepSeek showed more frequent positive differences in information completeness, clarity and empathy, and patient-family satisfaction, suggesting stronger performance in communication-related domains. Differences in medical accuracy and safety were more variable, with both positive and negative values across questions. These findings indicate that DeepSeek may support ICU family communication, but clinician oversight remains necessary for medically sensitive information involving accuracy and safety (Figure 3).

To further account for the clustered structure of the evaluation data, a mixed-effects ordinal logistic regression analysis was performed. The results were consistent with the primary analysis. DeepSeek responses had significantly higher odds of receiving better ratings in information completeness (OR = 2.005, 95% CI: 1.662–2.418, *p* < 0.001), clarity and empathy (OR = 1.996, 95% CI: 1.665–2.392, *p* < 0.001), and patient-family satisfaction (OR = 2.217, 95% CI: 1.844–2.666, *p* < 0.001). In contrast, no significant differences were observed in medical accuracy (OR = 1.046, 95% CI: 0.873–1.254, *p* = 0.623) or safety ratings (OR = 0.969, 95% CI: 0.801–1.172, *p* = 0.744) (Table 3). These findings further support the robustness of DeepSeek’s advantages in communication-related domains, while confirming its comparable performance with clinicians in medical accuracy and safety.

**Table 3:** Mixed-effects ordinal logistic regression analysis comparing DeepSeek and clinician responses across five evaluation domains.

Evaluation project	OR (95% CI)	*P*-value
Medical accuracy	1.046 (0.873–1.254)	0.623
Information completeness	2.005 (1.662–2.418)	<0.001
Clarity and empathy	1.996 (1.665–2.392)	<0.001
Safety assessment	0.969 (0.801–1.172)	0.744
Patient-family satisfaction	2.217 (1.844–2.666)	<0.001

OR, odds ratio; CI, confidence interval.

## 4 Discussion

To address persistent communication challenges in the ICU, this study compared responses from DeepSeek and clinicians to medical inquiries from patient-family members and assessed the impact of these responses on family satisfaction.

A total of 30 standardized consultation questions representing typical ICU communication scenarios were used to assess the quality of the responses. DeepSeek received higher ratings than clinicians in two domains, information completeness and clarity and empathy, and was associated with higher patient-family satisfaction. Moreover, across four evaluation metrics, including medical accuracy, information completeness, clarity and empathy, and patient-family satisfaction, DeepSeek consistently received more favorable ratings. However, these findings should be interpreted primarily as differences in perceived response quality under standardized written conditions, rather than as evidence of superior clinical competence.

DeepSeek’s strength in information completeness is particularly relevant in the ICU, where uncertainty and stress levels are high. Under such high-pressure conditions, clinicians may simplify explanations because of time constraints (28), potentially compromising communication quality, a critical determinant of family satisfaction (29, 30). In contrast, LLMs can provide structured and comprehensive explanations regarding disease progression, treatment rationale, and potential prognosis, without being affected by fatigue or workload (31, 32). This capability may address a common source of dissatisfaction: insufficient or inconsistent information during critical illness (33). In this study, DeepSeek consistently provided more thorough responses, which may help improve perceived clarity and reassurance.

The clarity and empathy demonstrated by DeepSeek are also notable (34), as these qualities have traditionally been considered uniquely human in critical care communication. However, emerging evidence indicates that well-designed AI systems can produce empathetic language and clear explanations (35). For instance, Ayers et al. reported that chatbot-generated responses to patient questions posted on a public social media forum were preferred over physician responses in 78.6% of evaluations and were rated significantly higher in both quality and empathy (21). In the ICU, where families frequently experience fear, uncertainty, and emotional exhaustion, DeepSeek’s ability to generate clear and empathetic responses supports its potential role as a supportive communication tool for both clinicians and families.

The observed higher scores in patient-family satisfaction further highlight the strong link between perceived communication quality and overall care experience. Recent studies have consistently identified effective communication as a primary predictor of family satisfaction and emotional adaptation during critical illness (36–38). Our findings support this association and suggest that AI-assisted communication may complement traditional clinician-family interactions by improving the perceived completeness, clarity, and emotional supportiveness of written explanations. However, because satisfaction was assessed using standardized written responses rather than real clinical encounters, these ratings may reflect textual preference or perceived response quality rather than true clinical satisfaction with bedside communication.

Further item-level and mixed-effects analyses strengthened the interpretation of the main findings. The heatmap of score differences across the 30 standardized ICU family questions showed that DeepSeek’s advantages were most consistently observed in communication-related domains, including information completeness, clarity and empathy, and patient-family satisfaction, whereas differences in medical accuracy and safety ratings were more heterogeneous. Similarly, the mixed-effects ordinal logistic regression analysis, which accounted for clustering within standardized questions, confirmed that DeepSeek was more likely to receive higher ratings in these communication-related domains, while showing no significant differences from clinicians in medical accuracy or safety ratings. Together, these analyses suggest that DeepSeek’s potential value in ICU family communication may lie primarily in enhancing the completeness, clarity, and perceived emotional support of written explanations, rather than replacing clinician expertise in medically sensitive domains.

However, higher satisfaction with AI-generated responses should be interpreted cautiously. LLMs may produce responses that are fluent, comprehensive, and empathetic in tone, but such qualities do not necessarily guarantee clinical correctness, contextual appropriateness, or safety in real-world decision-making. AI-generated responses may also contain uncertainties, omissions, or fabricated information, particularly when questions involve rapidly changing clinical conditions, complex prognostic judgments, or individualized treatment decisions. In addition, LLM outputs may reflect biases embedded in training data, including differences in language style, cultural assumptions, health literacy level, or communication norms. These factors could influence how family members perceive empathy and clarity and may partly explain favorable satisfaction ratings. Therefore, the apparent communication advantages of DeepSeek should not be interpreted as evidence that AI can replace clinician judgment or direct clinician-family communication in the ICU.

In the remaining evaluation domains, DeepSeek performed comparably to clinicians, suggesting that LLMs may have a useful knowledge base for generating general ICU-related explanations (39). This aligns with previous research on other medical LLMs, which reported knowledge levels comparable to or exceeding those of clinicians in specific areas (40). Nevertheless, comparable scores in a standardized evaluation should not be equated with autonomous clinical reliability. In practical ICU workflows, AI tools should be positioned as supportive systems rather than independent communicators. A feasible model would be to use AI to generate preliminary explanations, educational materials, or draft responses, which are then reviewed, edited, and delivered by clinicians. During such review, clinicians should not only verify medical accuracy and safety but also adjust response length, structure, and tone to fit the family member’s emotional state, health literacy, cultural background, and information needs. Clear escalation rules should be established so that questions involving prognosis, treatment decisions, end-of-life care, consent, or safety concerns are handled directly by clinicians. Future implementation should also include local validation, clinician training, education for patients and families, privacy protection, and continuous monitoring of AI-generated content to ensure accuracy, fairness, and safety.

## 5 Limitations

Although this study highlights the potential of DeepSeek to support ICU family communication, several limitations should be acknowledged. First, this was a standardized, vignette-based evaluation of written responses rather than a real-world clinical communication intervention. Therefore, it could not fully capture the emotional complexity, time pressure, nonverbal cues, real-time feedback, or interactive nature of actual ICU consultations. The findings should thus be interpreted as evidence of perceived written response quality under standardized conditions rather than direct evidence of bedside effectiveness. Second, the question set covered only selected ICU-related family consultation scenarios and included several perioperative and anesthesia-related topics, such as pain after anesthesia, fasting, and recovery time. These questions may reflect routine perioperative counseling rather than the most complex or ethically challenging ICU communication scenarios. Therefore, the results should not be generalized to discussions involving prognosis, end-of-life care, uncertainty management, treatment limitation, shared decision-making, or long-term outcomes. Third, AI-generated responses may have been rated more favorably because they were often longer, more structured, and more empathetic in tone than clinician responses. Such stylistic features may influence ratings of information completeness, clarity and empathy, and satisfaction, making it difficult to distinguish perceived communication quality from clinical content quality. Fourth, DeepSeek’s performance in individualized, ethically complex, or rapidly evolving clinical situations remains uncertain. LLMs may generate inaccurate, biased, unsupported, or overly confident information, especially when patient-specific judgment or real-time clinical interpretation is required. Finally, this study did not assess multi-turn conversations or shared decision-making. Future studies should use broader ICU scenarios, standardize response style, and evaluate clinician-supervised AI communication in real clinical settings.

## 6 Conclusion

DeepSeek demonstrated advantages in information completeness, clarity and empathy, and patient-family satisfaction under standardized written evaluation conditions. These findings suggest its potential to serve as a supportive tool for ICU family communication. However, clinician oversight remains essential, particularly for medically sensitive or individualized discussions. Future studies should validate clinician-supervised use of LLMs in real ICU settings.

## Data Availability

The original contributions presented in the study are included in the article/Supplementary material, further inquiries can be directed to the corresponding author.
