# Supplementary material for: An evaluation of DeepSeek and healthcare professionals’ Q&A capabilities in improving patient-family satisfaction in the ICU
Source: Front Med (Lausanne). 2026 Jun 8;13:1859158. doi: 10.3389/fmed.2026.1859158 (PMC13283868; doi:10.3389/fmed.2026.1859158)
Supplement: Supplementary file 1 [file Table_1.DOCX]

| **Table** | **Description** |
| --- | --- |
| **Supplementary Table 1** | **The CHART Checklist** |
| **Supplementary Table 2** | **The responses generated by DeepSeek and the clinicians** |
| **Supplementary Table 3** | **Baseline characteristic of patient and family** |

**Supplementary Table 1 The CHART Checklist**

| HEADING | # | CHART CHECKLIST ITEM | Page #* |
| --- | --- | --- | --- |
| Title & Abstract |  |  |  |
| Title | **1a** | State that the study is assessing one or more generative AI-driven chatbots for clinical evidence or health advice. | 1 |
| Abstract/Summary | **1b** | Apply a structured format, if applicable. | 1 |
| Introduction |  |  |  |
| Background | **2a** | State the scientific background, rationale, and healthcare context for evaluating the generative AI-driven chatbot(s), referencing relevant literature when applicable. | 2 |
|  | **2b** | State the aims and research questions including the target audience, intervention, comparator(s), and outcome(s). | 2 |
| Methods |  |  |  |
| Model Identifiers | **3a** | State the name and version identifier(s) of the generative AI model(s) and chatbot(s) under evaluation, as well as their date of release or last update. | 2 |
|  | **3b** | State whether the generative AI model(s) and chatbot(s) are open-source or closed-source/proprietary. | 2 |
| Model Details | **4a** | State whether the generative AI model was a base model or a novel base model, tuned model, or fine-tuned model. | 2 |
|  | **4b** | If a base model is used, cite its development in sufficient detail to identify the model. | 2 |
|  | **4c** | If a novel base model, tuned model, or fine-tuned model is used, describe the pre- and/or post-implementation/deployment data and parameters. | Not applicable |
| Prompt Engineering | **5a** | Describe the evolution of study prompt development. | 2-3 |
|  | **5ai** | Describe the sources of prompts. | 2-3 |
|  | **5aii** | State the number and characteristics of the individual(s) involved in prompt engineering. | 3 |
|  | **5aiii** | Provide details of any patient and public involvement during prompt engineering. | 3 |
|  | **5b** | Provide study prompts. | 2 |
| Query Strategy | **6a** | State route of access to generative AI model. | 2 |
|  | **6b** | State the date(s) and location(s) of queries for the generative AI-driven chatbot(s) including the day, month, and year as well as city and country. | 2 |
|  | **6c** | Describe whether prompts were input into separate chat session(s). | 2 |
|  | **6d** | Provide all generative AI-driven chatbot output/responses | 2 |
| Performance Evaluation | **7a** | Define the ground truth or reference standard used to define successful generative AI-driven chatbot performance. | 3 |
|  | **7b** | Describe the process undertaken for generative AI-driven chatbot performance evaluation. | 3 |
|  | **7bi** | State the number and characteristics of team members involved in performance evaluation. | 3 |
|  | **7bii** | Provide details of any patients and public involvement during the evaluation process. | 3 |
|  | **7biii** | State whether evaluators were masked to the identity of the generative AI-driven chatbot(s) under assessment. | 3 |
| Sample Size | **8** | Report how the sample size was determined. | 3 |
| Data Analysis | **9a** | Describe statistical analysis methods, including any evaluation of reproducibility of generative AI-driven chatbot responses. | 3-4 |
|  | **9ai** | Report the measures used for performance evaluation. | 3-4 |
| Results |  |  |  |
|  | **10a** | Report the alignment between generative AI-driven chatbot output and ground truth or reference standard using quantitative or mixed methods approaches as applicable. | 4 |
|  | **10b** | For responses deviating from the ground truth or reference standard, state the nature of the difference(s). | 4 |
|  | **10c** | Report the assessment for potentially harmful, biased, or misleading responses. | 4 |
| Discussion |  |  |  |
|  | **11a** | Interpret study findings in the context of relevant evidence. | 5-6 |
|  | **11b** | Describe the strengths and limitations of the study. | 5-6 |
|  | **11c** | Describe the potential implications for practice, education, policy, regulation, and research. | 5-6 |
| Open Science |  |  |  |
| Disclosures | **12a** | Report any relevant conflicts of interest for all authors. | 7 |
| Funding | **12b** | Report sources of funding and their role in the conduct and reporting of the study. | 7 |
| Ethics | **12c** | Describe the process undertaken for ethical approval. | 7 |
|  | **12ci** | Describe the measures taken to safeguard data privacy of patient health information, as applicable. | 7 |
|  | **12cii** | State whether permission/licensing was obtained for the use of original, copyrighted data. | 7 |
| Protocol | **12d** | Provide a study protocol. | Not applicable |
| Data availability | **12e** | State where study data, code repository, and model parameters can be accessed. | Not applicable |

*If in supplementary appendix, indicate “supp” and appendix #, if applicable.

Supplementary Table 2 The responses generated by DeepSeek and the clinicians

| **No.** | **Stage** | **Questioner** | **Question Content** | **DeepSeek** | **Clinicians** |
| --- | --- | --- | --- | --- | --- |
| 1 | Preoperative/  Intraoperative | Patient | Will I feel any pain after the anesthesia is administered? | No, that is not the case. The purpose of general anesthesia is to ensure complete loss of consciousness and memory during surgery. Anesthesiologists administer a series of medications to maintain the patient in a sleep-like state throughout the procedure, ensuring that no pain is felt. After surgery, as the anesthetics wear off, the patient may experience pain at the surgical site, which will be managed through a dedicated analgesic protocol. | General anesthesia induces sleep, preventing any pain perception during surgery. Spinal anesthesia may cause minor discomfort during injection, but the surgical site remains insensitive thereafter. Local anesthesia similarly ensures that the operative area is numb. Postoperative pain may occur as anesthetic effects wear off, and analgesic measures are proactively provided. |
| 2 | Preoperative/  Intraoperative | Patient | Am I allowed to wear dentures or contact lenses in the operating room? | Absolutely not. For your safety, all removable dental prostheses, contact lenses, jewelry, hairpins, and similar items must be removed before surgery. Dentures may dislodge under anesthesia and obstruct the airway, posing a risk of suffocation. Contact lenses can cause severe corneal injury while unconscious, leading to dryness, inflammation, or more serious complications. Please entrust these items to your family for safekeeping. | Removable dental prostheses may dislodge into the airway under anesthesia, and contact lenses can cause corneal hypoxia. All such items must be removed prior to entering the operating room and entrusted to family members for safekeeping, to be re-worn after recovery. |
| 3 | Preoperative/  Intraoperative | Patient | Will there be a significant amount of bleeding during the surgery? | Surgeons operate with meticulous precision to minimize blood loss. Simultaneously, anesthesiologists and the surgical team closely monitor vital signs, including blood loss, and administer blood or fluids as needed to maintain circulatory stability. You can trust in the team’s professional competence. | Preoperative coagulation assessment and intraoperative hemostatic techniques and medications control blood loss. Most surgical procedures involve minimal bleeding, which is well-managed and generally not a cause for concern. |
| 4 | Preoperative/  Intraoperative | Patient | How soon will I regain consciousness after the operation? | Recovery of consciousness depends on the type and duration of surgery, as well as the anesthetic agents used. Typically, patients regain consciousness within minutes to an hour after surgery. Initial grogginess and disorientation are normal during anesthesia recovery. Patients are transferred to the post-anesthesia care unit (PACU) or ICU for continuous monitoring until fully awake. | Recovery time depends on anesthesia type. General anesthesia typically requires 1–2 hours for awakening, while frail patients or prolonged procedures may take 3–4 hours. Patients are closely monitored in the recovery room. Other anesthesia methods usually maintain patient awareness throughout surgery. |
| 5 | Preoperative/  Intraoperative | Patient | Why is fasting required before surgery? What should I do if I feel hungry? | This is to prevent vomiting and aspiration during anesthesia. Swallowing reflexes are absent under anesthesia; if the stomach contains food or liquid, it may reflux into the trachea and lungs, causing aspiration or pneumonia. Although patients may feel hungry, adherence to fasting protocols is critical for safety. If thirsty, the lips can be moistened with a swab. Strict compliance with fasting instructions is essential. | Fasting is essential to prevent aspiration and subsequent pneumonia during anesthesia. If hunger is severe, patients should inform nursing staff, who may administer small amounts of intravenous glucose as needed. |
| 6 | Preoperative/  Intraoperative | Patient | Will a urinary catheter be inserted, and will it be painful? | For moderate to major or prolonged surgeries, a urinary catheter is typically placed to accurately record urine output, an important indicator of circulatory and renal function, and to prevent bladder overdistension. Placement occurs under anesthesia, causing no pain. Upon awakening, patients may experience mild discomfort or a foreign-body sensation, which is usually tolerable. The catheter is maintained for patency and hygiene, and removed as soon as clinically appropriate. | A urinary catheter is indicated for surgeries longer than two hours, procedures involving the urinary system, or when urine output monitoring is required. For general anesthesia, catheter insertion occurs after induction, causing no pain due to anesthesia and lubrication. Patients may feel mild discomfort postoperatively, and the catheter is removed once spontaneous urination resumes. |
| 7 | Preoperative/  Intraoperative | Patient | Will I be able to breathe on my own after surgery? | In most cases, spontaneous respiration gradually resumes as anesthetic effects subside. Immediately after surgery, while deeply sedated or under residual muscle relaxants, mechanical ventilation may be used temporarily to support breathing. Once respiratory strength and vital signs stabilize, the ventilator is removed to allow autonomous breathing. | Most patients can breathe independently postoperatively. Only a minority require temporary mechanical ventilation due to surgical or respiratory factors; the ventilator is removed once breathing is stable and consciousness is recovered. |
| 8 | Preoperative/  Intraoperative | Patient | What will happen if unexpected events occur during the operation? | Please rest assured that the operating room functions as a highly coordinated team environment. All potential emergencies are managed according to established protocols. Surgeons, anesthesiologists, and nurses are trained to rapidly identify and handle critical situations, ensuring patient safety. | Risk assessments and emergency protocols are established preoperatively. The operating room is staffed with specialized teams and equipped with emergency devices, ensuring immediate response to any unexpected events. |
| 9 | Preoperative/  Intraoperative | Patient | Can I see my family immediately after the surgery? | Upon immediate return to the ICU, patients require a brief stabilization period without visitors due to vital sign monitoring, device connections, and potential emergency interventions. Once stabilized, family visits are arranged according to ICU policy. Many ICUs also provide video visitation options. | ICU or recovery room observation is required before family visitation. Visits are scheduled according to institutional protocols for short durations until patient vital signs stabilize; extended visits are allowed once patients are transferred to general wards. |
| 10 | Postoperative/  ICU | Patient | Why do I experience a sore throat and difficulty speaking? | Postoperative sore throat, hoarseness, or phlegm sensation is common due to minor irritation from the endotracheal tube used to secure the airway during anesthesia. These symptoms typically resolve within one to two days. Communication can be facilitated using writing boards or gestures. | Postoperative sore throat is often caused by endotracheal tube irritation of the pharyngeal mucosa. Symptoms usually improve within 1–2 days. Patients are advised to avoid speaking loudly and may sip warm water as tolerated. |
| 11 | Postoperative/  ICU | Patient | How long will mechanical ventilation be required, and can it be discontinued? | Duration of mechanical ventilation depends on surgical type, anesthesia recovery, spontaneous respiratory strength, and pulmonary function. The goal is early extubation. When criteria are met—stable vital signs, effective spontaneous breathing, alertness, and satisfactory blood gas analysis—the anesthesiologist initiates extubation. Patient cooperation is critical for safety. | Duration of ventilatory support depends on respiratory recovery, ranging from 6–12 hours to 1–3 days. Daily assessments guide timely extubation, ensuring safe withdrawal of mechanical ventilation. |
| 12 | Postoperative/  ICU | Patient | Am I allowed to drink water or eat food after the procedure? | Oral intake is prohibited until full consciousness is regained and gastrointestinal function is confirmed, with no risk of vomiting. Intravenous fluids provide hydration and nutrition. Feeding begins with small amounts of water or liquid diet and gradually progresses to normal food based on tolerance. | Oral intake is permitted only after physician evaluation. Patients typically start with small amounts of water, progressing to liquid and normal diet based on tolerance. |
| 13 | Postoperative/  ICU | Patient | I feel pain — can additional analgesics be administered? | Effective analgesia is essential for recovery. Patients should not endure pain silently. Pain levels should be reported to nursing staff, who will administer analgesics as prescribed. Adequate pain control facilitates rest, coughing, sputum clearance, and early mobilization. | Pain management is feasible and encouraged. Patients report pain levels to nursing staff; mild to moderate pain is managed with oral or intravenous medications, while severe pain may require patient-controlled analgesia. Effective analgesia facilitates recovery. |
| 14 | Postoperative/  ICU | Patient | I feel dizzy or nauseous — is this normal? | Postoperative dizziness and nausea are common, resulting from anesthetic effects, surgical stress, analgesic side effects, or blood pressure fluctuations. Symptoms can be managed with medications, positional adjustments, or infusion rate modifications. | Postoperative dizziness and nausea are normal responses. Dizziness often results from blood pressure fluctuations, and nausea from anesthetic effects. These symptoms are monitored and treated as needed, usually resolving within 1–2 days. |
| 15 | Preoperative/  Intraoperative | Family Member | Where can family members wait, and can they receive updates at any time? | Hospitals provide designated waiting areas for family members to ensure timely communication. Surgical updates—including start, end, and critical milestones—are conveyed by the lead surgeon or anesthesiologist. Long procedures may involve periodic progress updates. Families should maintain communication availability. | Family members may wait in designated areas outside the ICU. Updates on intraoperative progress are provided proactively; urgent developments are communicated immediately. Nursing staff are available for inquiries. |
| 16 | Preoperative/  Intraoperative | Family Member | Is the surgery risky for elderly or physically frail patients? | We understand your concern. Advanced age or frailty increases perioperative risk. However, a comprehensive preoperative evaluation by multidisciplinary experts (surgery, anesthesia, ICU) is performed. Individualized anesthesia, surgical, and postoperative care plans are developed to minimize risk. Continuous monitoring ensures patient safety throughout the perioperative period. | Advanced age or frailty increases postoperative recovery challenges, but targeted monitoring and individualized care plans are in place to closely observe vital signs and maximize safety. |
| 17 | Preoperative/  Intraoperative | Family Member | Is anesthesia dangerous? Is there a risk of not waking up? | Modern anesthesia techniques are highly safe and mature. Anesthesiologists continuously monitor respiratory, cardiac, and hemodynamic parameters, adjusting medications as needed. The likelihood of failure to awaken due to anesthesia is extremely low; delayed emergence is typically related to patient factors such as age, comorbidities, or surgical trauma. The goal is safe and stable recovery. | Anesthesia carries some risk, but complications are rare. Preoperative assessments and continuous intraoperative monitoring, along with postoperative follow-up, ensure safe emergence from anesthesia. The likelihood of failure to regain consciousness is extremely low. |
| 18 | Preoperative/  Intraoperative | Family Member | What possible complications may occur? | All surgeries and anesthetic procedures carry potential complications, but preventive measures are implemented. Common risks include: cardiopulmonary events (arrhythmias, pulmonary infection, atelectasis), bleeding and infection (wound or internal), thromboembolism (deep vein thrombosis, pulmonary embolism), and other issues such as nausea, vomiting, or urinary retention. Disclosure is for informed consent, not prediction of occurrence. | Common risks include pulmonary infection, bleeding, and electrolyte disturbances. Real-time monitoring allows early intervention, minimizing potential complications. |
| 19 | Preoperative/  Intraoperative | Family Member | Will taking medication before surgery affect the procedure? | It is crucial to report all long-term medications preoperatively, especially antihypertensives, antidiabetics, and anticoagulants (e.g., aspirin, clopidogrel, warfarin). Some medications require continuation, others temporary cessation to prevent intraoperative bleeding. Physicians provide tailored guidance. Strict adherence is required. | Preoperative medications are recorded and evaluated. Most medications do not affect surgery; adjustments or surgical delays are implemented only when necessary. |
| 20 | Preoperative/  Intraoperative | Family Member | Does admission to the ICU mean the condition is critical? | ICU admission does not necessarily indicate critical illness; it signifies the need for enhanced monitoring and care, such as: postoperative stabilization of vital signs, temporary mechanical ventilation, and precise fluid and medication management. This ensures safe passage through the most critical recovery period. | ICU admission reflects the need for close monitoring and enhanced treatment, particularly for critically ill patients, ensuring timely stabilization. |
| 21 | Preoperative/  Intraoperative | Family Member | Who is the anesthesiologist, and will they remain present throughout the surgery? | Anesthesiologists remain at the patient’s side throughout the procedure, maintaining vital signs, managing respiration, circulation, and analgesia until safe transfer to PACU or ICU. | Anesthesiologists remain present throughout surgery, administering anesthesia, monitoring vital signs, and supervising postoperative emergence to ensure safety. |
| 22 | Preoperative/  Intraoperative | Family Member | When will the surgical results be available? | Once the main surgical steps are completed and patient condition is stable, the lead surgeon typically informs family members of intraoperative findings, procedures performed, and postoperative plans, usually after transfer to PACU or ICU. | After surgery, the lead surgeon informs family members of operative outcomes promptly. Definitive pathology results are usually available within 3–5 days and communicated when available. |
| 23 | Preoperative/  Intraoperative | Family Member | The patient feels anxious — is there any way to provide reassurance? | Preoperative anxiolysis may be administered with safe sedatives. Family support is also important; verbal encouragement and emotional stability help reassure the patient. | Patients are supported preoperatively with calming communication, adjustment of environmental factors, and, if necessary, mild sedatives. Family encouragement contributes to patient relaxation. |
| 24 | Postoperative/  ICU | Family Member | Is the patient awake now? Can they breathe spontaneously? | Emergence from anesthesia is gradual and may involve drowsiness or delayed response. Spontaneous breathing also recovers progressively. Mechanical ventilation is available in the ICU until the patient can breathe independently. Patience is essential during this process. | The patient is currently awake and capable of spontaneous breathing. Ongoing management is adjusted according to their condition, and any changes are communicated promptly. |
| 25 | Postoperative/  ICU | Family Member | Why is mechanical ventilation still required, and when can it be removed? | Postoperative mechanical ventilation supports oxygenation, reduces respiratory muscle workload, and stabilizes internal environment during anesthetic metabolism. Extubation is considered when consciousness, respiratory strength, circulatory stability, and blood gas analysis meet criteria. | Mechanical ventilation reduces respiratory workload. Extubation occurs once respiratory strength and oxygenation are adequate, guided by daily respiratory function assessments. |
| 26 | Postoperative/  ICU | Family Member | Why is visitation not allowed? Are there specific regulations for family visits? | ICU visitation is restricted to prevent infection, ensure uninterrupted care, and provide a quiet recovery environment. Fixed visiting hours or video options are available. | ICU visitation is restricted to reduce infection risk, typically allowing 1–2 brief visits per day, 1–2 persons per visit, according to posted schedules. |
| 27 | Postoperative/  ICU | Family Member | Are the patient’s vital signs currently stable? | ICU patients are continuously monitored for vital signs, with immediate intervention for any abnormality. Stable vital signs indicate that key parameters are within safe ranges. | The patient’s vital signs are currently stable overall, with heart rate, blood pressure, and oxygen saturation continuously monitored; any fluctuations are promptly addressed. |
| 28 | Postoperative/  ICU | Family Member | Why has the patient not regained consciousness? Has the effect of anesthesia not worn off? | Delayed emergence may result from residual anesthetic effects, surgical factors, or patient-specific conditions (e.g., comorbidities, electrolyte disturbances). Continuous monitoring and supportive measures facilitate recovery. | Delayed emergence may result from residual anesthetic effects or patient-specific factors. Consciousness is closely monitored, and most patients recover within hours. |
| 29 | Postoperative/  ICU | Family Member | Is there any sign of postoperative infection or bleeding? | Postoperative surveillance focuses on infection (temperature, blood counts, wound monitoring, prophylactic antibiotics) and bleeding (drainage volume, color, vital signs). Lack of special notification indicates condition remains controlled. | No signs of infection or bleeding have been observed. Continuous monitoring of temperature, laboratory values, wounds, and drainage allows rapid response to any abnormalities. |
| 30 | Postoperative/  ICU | Family Member | Is the patient in pain? What pain management measures are being provided? | Effective postoperative analgesia promotes recovery. Multimodal strategies may include patient-controlled analgesia, intravenous or oral medications, and regional nerve blocks. Pain is assessed through physiological indicators and patient response, with prompt management to ensure comfort during recovery. | Postoperative pain varies among patients. Analgesic regimens, including intravenous medications and patient-controlled analgesia, are adjusted according to patient response to minimize discomfort and support recovery. |

| **Supplementary Table 3 Baseline characteristic of patient and family** | | | |
| --- | --- | --- | --- |
|  | | Frequency (n) | Percentage (%) |
| **Patients** | |  |  |
| Age | 18-29 years | 69 | 11.5 |
|  | 30-39 years | 112 | 18.7 |
|  | 40-49 years | 158 | 26.3 |
|  | Above 50 years | 261 | 43.5 |
| Gender | Female, n (%) | 279 | 46.5 |
|  | Male, n (%) | 321 | 53.5 |
| Level of education | University level, n (%) | 73 | 12.2 |
|  | Non-university level, n (%) | 527 | 87.8 |
| History of surgery | Yes, n (%) | 243 | 41.5 |
|  | No, n (%) | 357 | 59.5 |
| ASA | III, n (%) | 231 | 38.5 |
|  | > III, n (%) | 369 | 61.5 |
| Type of disease | Respiratory failure, n (%) | 53 | 8.8 |
|  | Circulatory failure, n (%) | 115 | 19.2 |
|  | Septic shock, n (%) | 103 | 17.2 |
|  | Neurological crisis, n (%) | 83 | 13.8 |
|  | Metabolic disorders, n (%) | 145 | 24.2 |
|  | Postoperative or traumatic critical illnesses, n (%) | 39 | 6.5 |
|  | Other, n (%) | 62 | 10.3 |
| **Patient-family** | |  |  |
| Age (years) | 18-29 years | 39 | 6.5 |
|  | 30-39 years | 293 | 48.8 |
|  | 40-49 years | 197 | 32.8 |
|  | Above 50 years | 71 | 12.8 |
| Gender | Female, n (%) | 232 | 38.6 |
|  | Male, n (%) | 368 | 61.3 |
| Level of education | University level, n (%) | 58 | 9.7 |
|  | Non-university level, n (%) | 542 | 90.3 |
| Categorical variables are presented in frequency (%); Continuous non-normal data are presented in median (interquartile ranges). | | | |
